# The Impact of Formal School Entry on Children’s Social Relationships with Parents, Siblings, and Friends

**DOI:** 10.3390/children8100891

**Published:** 2021-10-06

**Authors:** Katharina M. Heuser-Spura, Julia Jaekel, Dieter Wolke

**Affiliations:** 1 Department of Pediatrics I, Neonatology, Pediatric Intensive Care and Pediatric Neurology, University Hospital Essen, University of Duisburg-Essen, 45122 Essen, Germany; 2 Faculty of Psychology, Ruhr-University Bochum, 44801 Bochum, Germany; 3 Unit of Psychology, Faculty of Education, University of Oulu, 90570 Oulu, Finland; Julia.Jaekel@oulu.fi; 4 Department of Psychology, University of Warwick, Coventry CV4 7AL, UK; D.Wolke@warwick.ac.uk; 5 Division of Mental Health and Wellbeing, Warwick Medical School, University of Warwick, Coventry CV4 7AL, UK

**Keywords:** parent–child relationships, sibling relationships, friendships, peer relationships, stability, developmental change, transitions

## Abstract

The normative transition to formal schooling confronts children with social challenges but also opportunities. Longitudinal research on how school entry impacts children’s family and friend-ship relationships is scarce. This study investigated social relationship qualities with parents, siblings, and friends among 1110 children (49.9% female) from the prospective, population-based Bavarian Longitudinal Study at 6 years (before school entry) and 8 years using a forced-choice card-sorting task. Multivariate analyses of variance revealed significant effects of age (i.e., school entry) on social relationship qualities with mothers (Pillai’s Trace (PT) = 0.28, *F*(9, 1101) = 47.73, *p* < 0.001), fathers (PT = 0.14, *F*(9, 1101) = 19.47, *p* < 0.001), siblings (PT = 0.27, *F*(9, 1101) = 46.14, *p* < 0.001), and friends (PT = 0.21, *F*(9, 1101) = 32.57, *p* < 0.001). On average, children reported higher levels of parental comfort after school entry. Companionable qualities increased in relationships with friends, whereas sibling relationships became more conflictual from preschool to early school age. Findings provide unique insights into how social relationships develop from preschool to early school age, supporting evidence of the growing importance of friends. Conflict was predominant and increasing in sibling relationships and should be considered more in future research.

## 1. Introduction

In middle childhood, parents, siblings, and friends are important close social relationships in children’s lives [1]. Experiences with these relationships substantially shape children’s development and adjustment, including mental health, behavior, academic outcomes, and social functioning [2,3,4,5,6]. Moreover, these different social relationships serve a variety of critical functions by fulfilling children’s social needs, for example, for caregiving, love, companionship, and support [7,8].

During development and with normative life events, children’s social relationships with parents, siblings, and friends and the social functions they serve change [8]. The transition to formal schooling is one life event most children face during middle childhood. School entry is associated with changes in children’s daily routines and social environments [9]. For instance, they spend more time away from home, having increasing contact with peers in the classroom or during leisure activities [10,11], which facilitates dyadic friendships. At the same time, children have to cope with more complex intellectual, behavioral, and social challenges [9,12,13]. Thus, school entry may be a time in which children may especially rely on the support of significant others, despite striving toward greater autonomy. Supportive, high-quality relationships with parents, siblings, and friends at preschool and early school age are crucial for children’s school success and also have enduring implications for later adjustment [3,4,6,14,15,16]. However, longitudinal research on how school entry specifically impacts children’s family and friendship relationships is scarce. Focusing on this normative transition will provide new insights into the changing roles of parents, siblings, and friends in fulfilling certain social needs.

Previous studies have consistently shown that relationships with parents change over the course of development [17,18,19]. According to attachment theory, children form an “affectional bond” of trust to their primary caregivers, mostly their mothers and fathers, when they care sensitively for their children’s physical and emotional needs [20,21,22]. Children explore the world from this “secure base” and seek proximity and comfort in times of distress [20,21,22]. As they grow up, children become more independent and adventurous. Accordingly, previous findings indicate that relationships with parents, on average, decrease in closeness (i.e., warmth, comfort) and support as well as increase in conflict from early to mid-adolescence [5,17,18,19]. However, there have been only few studies that specifically assessed the transition into formal schooling as a potential early marker of growing independence. These found that parent–child attachment and specific qualities, such as closeness and conflict, showed some consistency from preschool to early school age [23,24]. Thus, school entry may not substantially alter individual differences in perceived parent–child relationship quality. Moreover, previous studies indicated that parents, in particular mothers, were most important in providing comfort at early school age when children were sick or sad, although children spent increasing time with peers and preferred them as companions [22,25,26]. Mean-level analyses yielded that mother–child closeness and conflict reported by mothers did not change from preschool age to first grade, whereas fathers experienced an increase in closeness across the transition to formal schooling [23]. However, it is uncertain whether the challenging event of school entry may actually increase rather than decrease children’s need for parental closeness and comfort.

Both siblings and friends are prominent social partners in middle childhood [27,28]. Sibling relationships constitute a lifelong, involuntary relationship and vary substantially in quality, with most dyads experiencing both times of warmth and conflict [8,29,30]. In contrast, friendships are usually voluntary, based on mutual liking and trust, and characterized by reciprocity and egalitarian exchanges [8,11,31]. Most children in Western countries have at least one sibling living in their household [6,32], whom they spend a substantial amount of time with, especially during early childhood [33]. Thus, siblings can be great play companions and may provide an important context for intimacy and disclosure [34,35]. In addition, siblings are of different age and interactions usually include hierarchical social roles. Older siblings may take care of their younger siblings and serve as teachers and sources of advice [34,36] or are more likely perpetrators of aggression against their younger siblings [37,38].

Especially when children enter school, peers become an increasingly important part of their daily lives. Most children in Western countries have at least one friend [11,28] and by early school age often spend more time with friends than with siblings [39,40]. Previous studies have indicated developmental change in sibling and friendship relationships from early school age into adolescence. For example, relationships with siblings seem to become increasingly egalitarian, whereas levels of conflict, affection, companionship, and support may decrease [41,42,43]. In contrast, friendships were rated as increasingly companionable and supportive [26,44,45], exceeding the importance of siblings by adolescence [46].

However, longitudinal research on how school entry may change relationships with siblings and friends is scarce. It has been found that preschool- and early school-aged children were more likely to turn to friends than to siblings to satisfy companionship needs, such as playing or having fun [22,25,47]. However, it is unknown whether this gap between siblings and friends increases when children enter formal schooling. On the other hand, previous studies indicated moderate individual stability in maternal reports and observations of positive and negative sibling behavior from preschool to early school age [14,48]. Similarly, kindergarten children with high-quality friendships as reported by mothers scored significantly higher on later friendship quality in first and third grade than children with no friends, average, or low-quality friendships [4]. However, previous studies did not provide a differentiated picture of stability and change in different social relationship qualities, including companionship, rather than global quality.

Conflict has been found to be a more salient feature in sibling than in friendship relationships at preschool and school age [26,49,50]. Mothers reported a decline in levels of sibling negativity, such as arguing and physical fights, and conflicts with friends from preschool to early school age [51,52]. In contrast, sibling aggression, including bullying and victimization, showed a peak at early school age and declining prevalence rates across school years [53]. Future studies are warranted to better understand changes in interaction negativity and conflict among siblings and friends.

Previous longitudinal research on children’s relationships with parents, siblings, and friends across the transition to formal schooling exhibited some limitations. Some studies investigated certain social relationships in isolation and often focused only on selected qualities [4,14,23,51,52]. Other studies analyzed composite scores [4,14,48], which restricts detailed investigation of different qualities. Finally, some studies were based only on parent reports [4,14,23,51,52]; however, especially when children enter school, parents may have less knowledge about their social experiences outside home [47].

The current longitudinal study expands upon previous research by investigating how the normative event of school entry impacts children’s family relationships with parents and siblings and their friendships in a prospective, epidemiologic cohort using a natural experimental design. In addition, this study provides a differentiated picture of stability and change by considering multiple social relationship qualities and focusing on children’s own perspectives. Using a social network approach [7], this study allows a direct comparison of the different roles that parents, siblings, and friends play in children’s lives across the transition to formal schooling. Finally, by using a forced-choice card-sorting task, our findings will improve our understanding of the relative importance of different social relationships in fulfilling certain social needs.

To this end, children were asked about their perceptions of different social relationship qualities with mothers, fathers, siblings, and friends before they entered elementary school (at 6 years of age) and at early school age (8 years of age). First, we expected mean-level changes of social relationship quality from age 6 to 8 years for all social relationship types. Specifically, we examined whether children’s perceptions of parental comfort increased from preschool to early school age. We investigated changes in companionable qualities, such as having fun, playing, and prosocial behaviors as well as conflicts in relationships with siblings and friends. Second, we examined whether individual differences in perceptions of social relationship quality with mothers, fathers, siblings, and friends remained stable from preschool to early school age despite hypothesized mean-level changes over time. Specifically, we tested to what extent social relationship qualities at 6 years predicted these qualities two years later. Finally, we considered the effects of individual differences on children’s social relationships at 6 and 8 years of age. Children born preterm (i.e., <37 completed weeks of gestation) have been found to face an increased risk of social relationship difficulties, especially with peers [54,55,56,57,58,59]. Moreover, there is evidence that child sex and family socioeconomic status (SES) contribute to differences in social relationship qualities [19,26,41,44,46,60,61], and thus, these factors were included in analyses.

## 2. Materials and Methods

### 2.1. Participants

**Figure 1:**
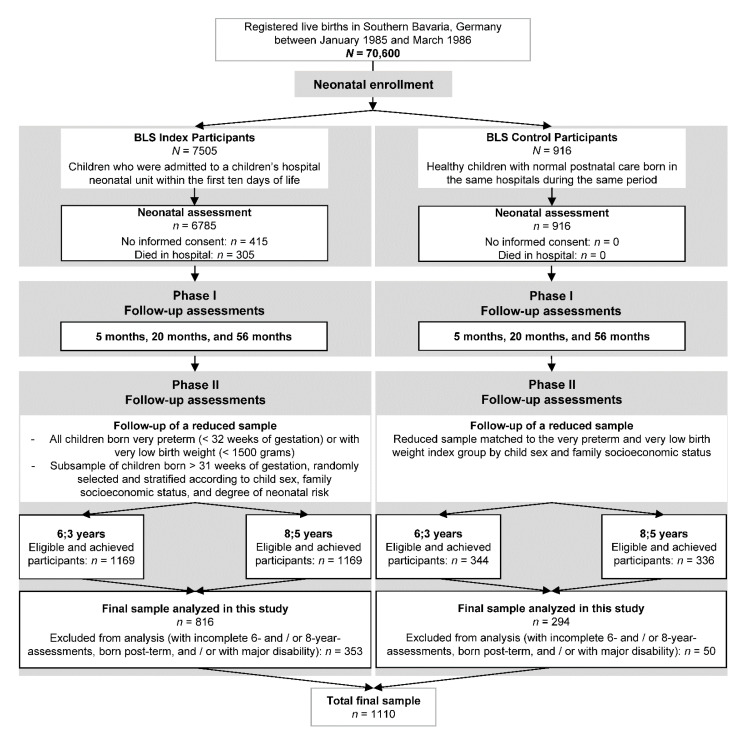
Flow-chart of participants. Flow of participants through the first two phases of the Bavarian Longitudinal Study until the age of 8 years (adapted from [64]). BLS, Bavarian Longitudinal Study.

Children were assessed as part of the prospective Bavarian Longitudinal Study (BLS), which is a geographically defined, population-based sample of neonatal at-risk children born between January 1985 and March 1986 in Southern Bavaria (Germany). Of 70,600 registered live births, 7505 children (10.6% of all live births) who were admitted to children’s hospitals within the first ten days after birth were recruited. Additionally, 916 healthy control children born at term in the same obstetric hospitals during the same period were included [62]. Details of the sampling criteria, design, and dropout rates have been described previously [63]. Figure 1 displays a flow diagram of participants through the first two phases of the BLS until age 8 years.

The full initial sample (*N* = 8421) was studied from birth to 56 months of age. A reduced sample of 1513 children was selected for Phase 2 follow-up assessments at 6 and 8 years of age. This sample included all children born very preterm (<32 weeks gestational age (GA)) or with very low birth weight (<1500 g), and a subsample of children born >31 weeks of gestation, randomly selected and stratified according to child sex, family SES (low, middle, high), and degree of neonatal risk (none, low, moderate, high) [64]. Non-German speaking children (*n* = 43) were excluded because interviews, cognitive assessments, and behavioral ratings could not be administered. Of this reduced eligible sample (*n* = 1513), 1184 children with complete assessments at both measurement points on the Card-Sorting Task of the Friendship and Family Interview [65,66] were included. Children born post-term (>41 weeks GA; *n* = 33) were excluded due to their risk for adverse developmental outcomes [67]. Furthermore, children who suffered from major disability (*n* = 41) were not included in the analyses. Major disability was defined as having a diagnosis of cerebral palsy (grade 3: walking not possible, but crawling; or grade 4: no active movement possible), blindness, hearing impairment (deaf; not or insufficiently corrected), or an IQ < −2 standard deviations compared to the standardized mean of a normative sample (i.e., <70) [62]. Of the included 1110 children (gestation range: 25–41 weeks), most (*n* = 705, 63.5% of the final sample) were born at term (37–41 weeks GA), while 250 (22.5%) were born moderately or late preterm (32–33 weeks GA, 34–36 weeks GA, respectively), and 155 (14.0%) were born very preterm (<32 weeks GA).

### 2.2. Procedure

During their child’s admission to the neonatal unit or postnatal ward, parents were approached within 48 h, and the study aims were explained to them. Children whose parents had given written informed consent for their child to participate (98% of those approached) were included in the study. Prenatal data was drawn from medical records in the obstetric units, whereas peri- and neonatal data was assessed prospectively with standardized interviews and medical examinations. Information about social and family background was collected using structured interviews performed within the first ten days of the child’s life. Data collection at the 6- and 8-year follow-up assessments was administered by a multidisciplinary study team of trained pediatricians, postgraduate clinical psychologists, and psychometric assistants who were blind to child and family characteristics. At the 6-year assessment, in accordance with standard school entry age in the state of Bavaria (Germany), 92.3% of the children had not entered elementary school, whereas 7.7% had been in school for less than three months. At the 8-year assessment, most children were in second grade of elementary school. Neurological and cognitive assessments, behavior ratings, and child and parent interviews were performed within one whole day [63,68,69]. Ethical approval of the study was provided by the Ethics Committee of the University of Munich Children’s Hospital and the Bavarian Health Council in Germany (Landesärztekammer). The study was conducted in accordance with the ethical principles defined in the Declaration of Helsinki.

### 2.3. Measures

#### 2.3.1. Biological and Medical Variables at Birth or Neonatal

Information about biologically determined child sex, GA, and birth weight was taken from medical records.

#### 2.3.2. Social Variables at Birth

Family SES was assessed with a structured parental interview within the first ten days after birth. Family SES was computed as a weighted composite score based on maternal and paternal highest educational qualification as well as occupation of the self-identified head of the family (usually father or mother), according to Bauer [70]. The three scores obtained were summed up, averaged, and then grouped into three categories (1 = low, 2 = middle, 3 = high SES).

#### 2.3.3. Perceived Social Relationship Qualities at 6 and 8 Years of Age

The Card-Sorting Task was administered as part of the semi-structured Friendship and Family Interview [65,66] to assess children’s perceptions of the quality of their social relationships with mothers, fathers, siblings, and friends at age 6 and 8 years. First, children were asked to list the people who belonged to their family (i.e., mother, father, and siblings); then, they were asked to list playmates and friends (for detailed information, see Figure S1 in the Supplementary Materials online). Next, children were instructed to choose Playmobil^®^ figures who represented their mother, father, siblings, and friends. The Playmobil^®^ figures were placed on a board that had a posting slit below each social relationship group. The Card-Sorting Task contained 36 cards each depicting a statement with a positive or negative feeling or action that had either originated from the child (e.g., “Who do you most like to cuddle with?”, “Who do you not at all like to cuddle with?”) or from the other person (e.g., “Who likes to cuddle with you?”, “Who sends you away when you want to cuddle?”). The order of items was quasi-randomized and fixed for all children (for detailed information, see Figure S1 in the Supplementary Materials online). The statements on the cards were read out loud. Then, the children were asked to assign each respective card to one of the persons on the board using a forced-choice response format: (a) mother, (b) father, (c) siblings, or (d) friends. If they felt that a card did not apply to any of the available groups, it could be posted to a building block representing nobody that was also placed on the board. Responses for each card were coded numerically according to social relationship type (i.e., mother = 1, father = 2, siblings = 3, friends = 4, nobody = 5, respectively; for detailed information, see Figure S1 in the Supplementary Materials online). The task consists of nine subscales, each tapping into another social relationship quality or need (i.e., Care, Comfort, Bad Conscience/Trust, Fun/Fooling Around, Playing, Prosocial Behavior, Cuddling, Conflicts, Affection) with four items (i.e., two positive and two negative), respectively. A list of all 36 items is shown in Table S1 (see Supplementary Materials online). For each of the nine subscales, the difference between positive and negative cards (i.e., number of positive cards assigned to person—number of negative cards assigned to person) was calculated to obtain valence scores for each social relationship type at 6 and 8 years of age, respectively. These valence scores ranged from −2 to +2, with higher values (i.e., positive values) indicating more positive than negative cards, lower values (i.e., negative values) indicating more negative than positive cards, and 0 indicating either no cards were assigned to this person or the same number of positive and negative cards. Social relationship quality subscales Fun/Fooling Around, Playing, and Prosocial Behavior were moderately to highly correlated (see Table S2 in the Supplementary Materials online) and averaged separately for social relationships with siblings and friends at 6 and 8 years of age to obtain companionship composite scores, respectively. These ranged from −2 to +2. Interviewers were trained over two months to ensure reliability and validity. All interviews were videotaped and double-rated by two psychologists. Interrater reliability was excellent with Cohen’s kappa > 0.95.

### 2.4. Statistical Analyses

Analyses were performed using SPSS 26.0 (IBM SPSS Statistics for Windows, IBM Corp., Armonk, NY, USA). Prior to the main analyses, descriptive statistics were calculated, and the unique effects of control variables child sex, family SES, and GA on children’s social relationship qualities at 6 and 8 years of age were determined. Curve estimations for the effect of GA on different valence scores indicated that overall, linear models provided the best fit. Multiple linear regressions were run to explore whether child sex (0 = male, 1 = female), family SES (dummy-coded), and GA (25 to 41 weeks) predicted valence scores of children’s social relationship qualities at age 6 and 8 years, adjusting for school entry status at 6 years (0 = not in school, 1 = in school). Repeated-measures one-way multivariate analyses of variance (MANOVAs) were conducted to examine the effects of age (6 years vs. 8 years) on social relationship quality with the different subscales as dependent variables. Separate analyses were performed for social relationships with mothers, fathers, siblings, and friends. To determine the stability of individual differences, multiple linear regressions tested to what extent valence scores at 6 years predicted valence scores at 8 years of age, after controlling for child sex, family SES, GA, and school entry status at 6 years. To avoid inflation of type 1 error, Bonferroni–Holm correction was used, adjusting the alpha level (initially set at *p* < 0.05) for multiple testing, which was two-tailed for all analyses. Bias-corrected and accelerated confidence intervals were based on 5000 bootstrap samples.

## 3. Results

### 3.1. Sample Description

Table 1 summarizes the descriptive characteristics on biological, medical, and social variables of the study sample. About half of the children were female (49.9%), the mean GA was 36.89 weeks (*SD* = 3.75), and the mean birth weight 2727.35 g (*SD* = 906.40). Most of the children were singletons (92.1%) born >36 weeks of gestation (63.5%). Most of them were living in two-parent households (94.7%) with medium or high SES (70.5%). More than half of the children (67.6%) lived in rural areas of Bavaria.

### 3.2. Preliminary Analyses—Effects of Child Sex, Family SES, and GA on Valence Scores at 6 and 8 Years of Age

Multiple regression models tested the unique effects of child sex, family SES, and GA on valence scores at 6 and 8 years of age for different social relationship qualities and types, corrected for school entry status at 6 years. Analyses were adjusted for multiple testing using Bonferroni–Holm correction, and three effects survived this correction. Boys cuddled less with their fathers than girls did at 6 and 8 years (6 years: *R*^2^ = 0.02, *B* = 0.18, *BCa* 95% *CI* (0.09, 0.27); 8 years: *R*^2^ = 0.03, *B* = 0.25, *BCa* 95% *CI* (0.15, 0.35)). Moreover, children born at lower GA reported having less fun and fooling around less often with friends at age 8 years (*R*^2^ = 0.03, *B* = 0.03, *BCa* 95% *CI* (0.02, 0.05)). Family SES showed no significant relationship to any of the social relationship qualities.

### 3.3. Stability and Change in Valence Scores from Preschool to Early School Age

Table S3 (see Supplementary Materials online) shows that the most positively rated qualities for mothers were Care, Comfort, and Cuddling, whereas fathers had lower scores and Bad Conscience/Trust was evaluated as most negative. Overall, most scores calculated for sibling relationships indicated negative valence, in particular for the subscales Conflicts and Comfort. Ratings for friends revealed that valence scores for the subscales Fun/Fooling Around, Conflicts, Playing, and Prosocial Behavior were most positive, whereas Comfort was most negative.

**Figure 2:**
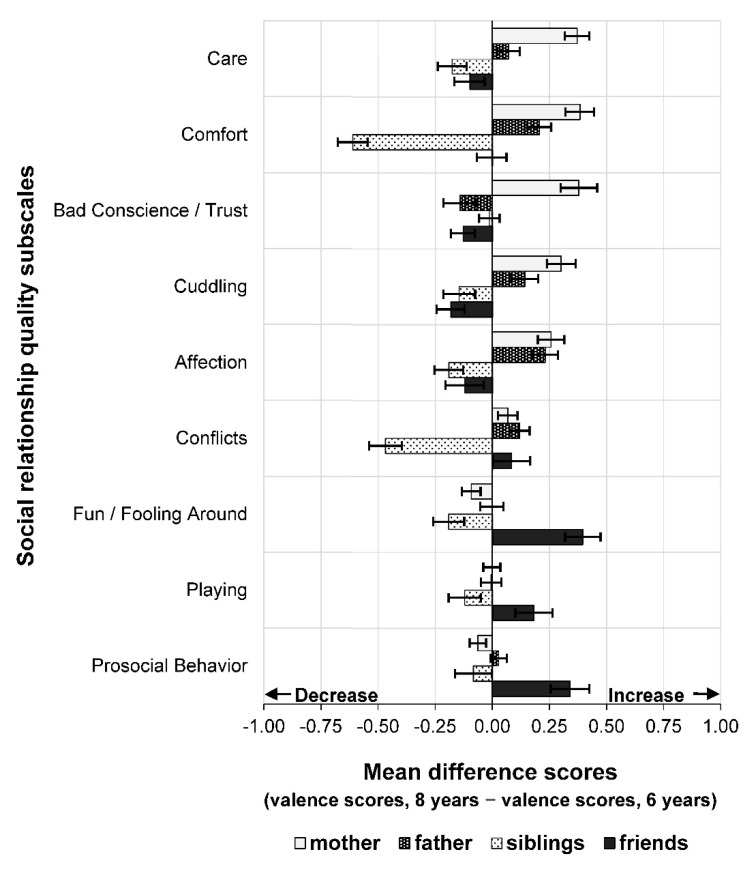
Mean-level changes in social relationship quality subscales between 6 and 8 years according to social relationship types (*N* = 1110). Mean difference scores were obtained by subtracting valence scores at 6 years from valence scores at 8 years (i.e., Time 2 minus Time 1). Error bars represent 95% confidence intervals. The subscale Conflicts is inverted, negative values indicate an increase in conflicts and positive values indicate a decrease in conflicts.

**Figure 3:**
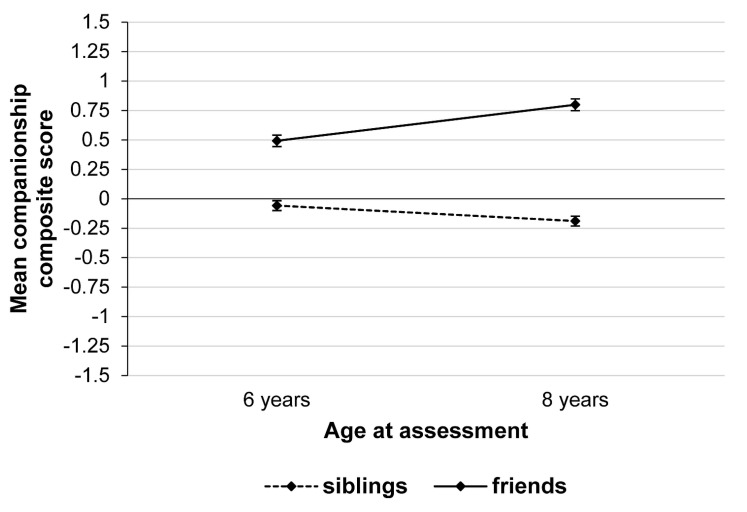
Mean companionship composite scores for social relationships with siblings and friends at 6 and 8 years (*N* = 1110). Error bars represent 95% confidence intervals. Companionship composite scores were obtained by averaging subscales Fun/Fooling Around, Playing, and Prosocial Behavior separately for social relationships with siblings and friends at 6 and 8 years of age. Companionship composite scores ranged from −2 to +2.

Repeated-measures one-way MANOVAs were carried out to examine the effects of age (6 years vs. 8 years) on children’s social relationship quality with the different subscales as the dependent variables. Separate analyses were run for social relationships with mothers, fathers, siblings, and friends. As expected, there were significant effects of age as shown in Table 2. Figure 2 indicates that on average, valence scores of most qualities in relationships with parents increased from age 6 to 8 years, in particular the subscales Care and Comfort for mothers and Affection and Comfort for fathers. In contrast, for siblings, most valence scores decreased from preschool to early school age, in particular the subscales Comfort and Conflicts. Card sorting with regard to friends showed a heterogeneous pattern of mean-level stability and change with subscales Fun/Fooling Around, Playing, and Prosocial Behavior increasing, and subscales Cuddling, Bad Conscience/Trust, Care, and Affection decreasing from age 6 to 8 years (for details, see Table S3 in the Supplementary Materials online). Figure 3 displays mean-level changes in companionship composite scores between 6 and 8 years of age that were calculated by averaging subscales Fun/Fooling Around, Playing, and Prosocial Behavior. Overall, companionship decreased in relationships with siblings and increased in friendships from age 6 to 8 years.

Multiple regression models revealed that higher valence scores at 6 years predicted higher valence scores at 8 years of age, irrespective of social relationship type and quality (except for subscale Cuddling for friends). As shown in Table 3, positive associations were particularly evident for the subscales Cuddling, Comfort, and Bad Conscience/Trust in social relationships with mothers and fathers, for the subscales Conflicts and Affection in sibling relationships, and for the subscales Playing, Care, Prosocial Behavior, and Fun/Fooling Around in friendship relationships. Almost all models survived the correction for multiple testing, except for the subscales Conflict and Prosocial Behavior rated for mothers, subscales Playing and Prosocial Behavior for fathers, and subscale Bad Conscience/Trust for friends.

## 4. Discussion

This prospective population-based study investigated how formal school entry impacts children’s perceptions of the quality of their social relationships with parents, siblings, and friends using a natural experimental design. Overall, mean-level analyses yielded significant changes in social relationship qualities with mothers, fathers, siblings, and friends from age 6 to 8 years, as expected. Specifically, children reported higher levels of parental comfort by early school age, especially from mothers. Moreover, friendships became more companionable, whereas companionship in relationships with siblings sharply decreased from preschool to early school age. Conflict was predominant in relationships with siblings, and interactions became even more conflictual after children had entered school. Overall, higher valence scores at 6 years predicted higher valence scores at 8 years of age, indicating modest individual stability in children’s perceptions of most social relationship qualities, in particular for trust, comfort, and emotional closeness (i.e., cuddling) in relationships with parents, affection, and conflict in sibling relationships, and companionable qualities in friendships. After correcting for multiple testing, only child sex and GA showed small associations with social relationship qualities. Family SES was not related to any social relationship qualities.

Findings of this study extend our understanding of how children perceive their family and friendship relationships across the normative transition to formal schooling. School entry marks substantial changes in children’s social environments and psychological goals [9,12], in which peers become increasingly important and preferred companions [10,25]. Previous studies showed that parents, in particular mothers, were still most important in providing comfort and emotional support after children had entered formal schooling [22,23,25]. However, findings of this study expand upon previous research [23,25] suggesting that children’s perceptions of parental comfort and affection do increase from preschool to early school age. Thus, the first years of formal schooling, in which children have to cope with new social and academic challenges [9,12] may be a time of heightened need for support from parents. This is a novel result with important practical implications for parents and teachers. Parents should particularly support their children during their first years of formal schooling, also considering that this period has long-term implications for children’s development and adjustment, including academic achievement [9,15].

In addition, results of this study support a larger child-perceived role of mothers in providing care and comfort for their children [25,71]. Nevertheless, the role of fathers in the children’s perceptions of affection and comfort also increased from preschool to early school age. During the last decades, there have been increasing rates of maternal employment in Western industrialized countries [72], including Germany [73], but most mothers still spend more time with children than fathers, in particular with caregiving tasks [71]. This cannot be attributed to fathers being absent, as in this population, most of the parents were living together (6 years: 89.9%; 8 years: 87.5%). Thus, the findings of this study indicate perceptions of different roles of mothers and fathers that children experienced in this predominantly rural, Catholic sample with traditional co-habitation. Nevertheless, future studies are needed that address whether increasing paternal involvement in children’s lives [74] may affect their perceptions of social relationship quality.

Findings further improve our understanding of how school entry changes children’s social relationships with siblings and friends. Consistent with previous studies at preschool, early school age, and in adolescence [7,22,25,40,47], children seem most likely to turn to friends for companionship, such as having fun, playing, and sharing, whereas siblings received lower ratings on these dimensions. In Germany, children usually attend day-care outside their homes before age 6 years (96.9% of children in this study), which facilitates daily contact with other, non-related children, whereas interaction time with siblings has been found to decrease throughout middle childhood [39]. Moreover, children reported increasing levels of companionable qualities and prosocial behavior in their relationships with friends from age 6 to 8 years, whereas for siblings, companionship decreased. Thus, overall, school entry may heighten the gap between siblings and friends. This supports the growing importance of friends as preferred companions during middle childhood [25], spending more time with them at school or during leisure activities [10].

Friendships play a critical role in supporting children’s adjustment to school [3,4]. Therefore, it is important that teachers facilitate the formation of friendships and encourage children’s social skills in the classroom. In particular during the first months after school entry, teachers may provide frequent opportunities for social interaction and contact with peers [75], and offer activities in small groups [76,77]. In addition, teachers may include classroom programs that focus on improving social skills and friendships [78,79].

Our findings are in accordance with children’s changing conceptions of friendships as they grow up [11,31]. In early childhood, positive affect and play are predominant characteristics of friendships, whereas throughout middle childhood, sharing and helping behaviors increase, which is a normative change accompanying prefrontal cortex maturation and theory of mind development [80]. However, when children enter school, they may lose some preschool friends, while forming new friendships [81], which may explain decreases in mean-levels of trust and emotional closeness (i.e., cuddling) found in this study. 

Conflict turned out to be most predominant in relationships with siblings, whereas, on average, children reported to argue rarely with friends. This is in line with previous studies on preschool-aged children and adolescents [7,49,50]. Moreover, consistent with longitudinal studies across early school age [26,41], interactions with siblings became even more conflictual from age 6 to 8 years. This contrasts previous findings indicating declines in maternal reports of sibling negativity, such as arguing and physical fights, from preschool to first grade [51]. It seems crucial to consider both children’s and their parents’ perspectives when investigating social relationships within the family. Sibling relationships are commonly described as emotionally intense, and most children experience both warm and conflictual qualities [6,29]; however, in some sibling dyads, negativity, conflicts, and rivalry dominate [29]. Overall, research on changes in sibling conflict, especially across the transition to school, is scarce [14,48,51]. Results of previous studies on the longitudinal course of sibling relationships from school age to adolescence varied depending on whether children had older or younger siblings and according to sex constellations [41,48]. For example, younger siblings perceived higher levels of conflict than older siblings did during the first years of school [41]. In addition, sibling intimacy decreased for mixed-sex dyads from middle childhood to early adolescence, whereas same-sex dyads showed stable trajectories [41]. Our current findings show that sibling relationships became less comforting after children had entered school, which adds to evidence of decreases in support found from early school age to early adolescence [26,43]. Deteriorating relationships with siblings may be due to children’s increasing interest in connecting with peers and forming new friendships when entering school [11,25,45,48,81]. Children reported to preferably play with same-sex peers [48,81,82], while interest for their other-sex siblings decreased [48]. Especially boys preferred to play with male peers and had less interest in their younger sisters [48]. Thus, changes in sibling relationships may occur as they give room to a growing importance of friends in children’s lives. Future studies are needed to investigate changes in the quality of sibling relationships from preschool to early school age while considering effects of birth order and sex constellation, information that was not available in this study. Although sibling relationships may become increasingly egalitarian throughout adolescence [26,83], siblings still perceive an imbalance of power at early school age [42,83]. Older siblings were reported to be more dominant [42,84], while younger siblings start standing up to their older siblings in accordance with advances in their social competence, which may increase sibling conflict [83]. The perception of power asymmetry between siblings also plays a critical role in sibling bullying [30,37]. Sibling bullying is one extreme form of sibling aggression, which intends to cause harm, is characterized by a power difference, and occurs frequently over time with prevalence rates up to 50% [30]. Previous studies found that sibling bullying and victimization increase until early school age [53], which is consistent with the findings of this study on sibling conflict. Thus, parents and teachers should give particular attention to sibling conflict and aggression given the substantial impact on children’s mental health, self-worth, and social adjustment [29,30], in particular as those bullied by siblings are also more often bullied at school and effects are cumulative on mental health [85]. Sibling relationship problems are often under-recognized, normalized, and not considered in the treatment of mental health problems. This study provides further evidence that sibling relationships are, on average, the most conflictual and should be considered more in future research [37,38].

The current findings also indicate modest individual stability in children’s social relationships from preschool to early school age. For parent–child relationships, specifically, perceptions of trust, emotional closeness (e.g., cuddling), and comfort were moderately associated with perceptions of these qualities two years later. This may indicate some consistency in attachment-related interactions across the transition to school [22,25], which has also been found in previous studies [23,24,86]. For siblings, the strongest associations from preschool to early school age were found for affection and conflict, which represent two important indicators of sibling relationship quality [29]. This is consistent with a recent study on positive and negative sibling behavior [14]. Given that children with more conflictual sibling relationships at preschool age may still have more conflicts with their siblings after they have entered school, it seems crucial that parents pay special attention to their children’s negative interactions and consider intervening early. With regard to friendships, companionable qualities such as sharing toys, having fun, and playing showed some individual stability. These reflect age-appropriate features of friendships in middle childhood, such as enjoyment, positive affect, playing, and sharing toys. Many children keep some of their preschool friends despite attending a new school, which may facilitate maintaining continuity in friendship behaviors [4,81]. In contrast, other features, such as emotional closeness and trust, which have been found to become more important friendship features at later ages [31], showed less individual stability. Overall, this study provides a differentiated picture of individual stability and change in children’s social relationships across the transition to formal schooling.

The current findings provide little evidence that child sex, family SES, and GA play a critical role in children’s perceived social relationship qualities at 6 and 8 years of age. Most associations of child sex with social relationships did not survive correction for multiple testing. This is in line with previous studies indicating that sex differences may be less prominent at preschool or early school age [3,48,52] and potentially become more noticeable after middle childhood [19,26,41,44]. However, this study found that girls’ relationships with their fathers were more physically close (i.e., more cuddling) than for boys, although differences were small. Thus, findings extend previous research [23] suggesting more closeness among fathers and daughters across the transition to school based on children’s own perceptions in addition to parent reports. It has been also demonstrated that social disadvantage, such as low family income, is associated with poorer parent–child and peer relationships at early school age [60,61]. However, overall, children of lower SES perceived similar quality of social relationships as those who were socially more advantaged in this study at preschool and early school age. Health care and social security in Germany is universal and may reduce the disadvantages seen in samples drawn from societies with larger social inequality [87]; thus, may have eliminated differences in social relationship quality according to SES in this study. Finally, in line with previous findings in infancy [59,88], children born preterm did not perceive the relationship with their parents as poorer than term born children by middle childhood, despite a different start into life [89]. Furthermore, no significant associations were found between GA and sibling relationship quality. Children born at lower GA reported experiencing less fun with friends at age 8 years; however, there were no significant associations between GA and other qualities of their friendship relationships. However, social relationships remain a major concern in preterm follow-up research [54,55,56,58,90,91,92]; thus, future studies should investigate trajectories of different social relationship facets from childhood into adulthood adopting a multimethod approach and considering different degrees of GA [90,91].

Strengths and Limitations

The strengths of this study comprise data collection from a large, prospective, whole-population cohort, which was followed longitudinally at 6 and 8 years of age, including an important normative transition from preschool into school. Moreover, children’s own perceptions of their social relationship qualities were assessed using standardized interviews, whereas many previous studies asked their parents [4,14,23]. In line with previous studies at preschool age [47,49], children’s nominations were different for different social relationship qualities and types in this study, which may indicate that they were able to distinguish between different qualities. Comparability in assessments across ages was ensured by using the same instruments at both measurement points. The effect of formal school entry on children’s social relationships was examined using a natural experimental design with one group. Furthermore, the current study presented an interview for assessing children’s social relationship qualities that identifies which member of their social network is most important and first choice in satisfying certain needs [65,66]; information that was masked by previous designs [7]. The Card-Sorting Task has good face validity (i.e., children assign different cards to the respectively best-fitting person) and provides a broad view of children’s different social relationships. The playful character (i.e., card-sorting game with play figures and a board with posting slits), the forced-choice response format, and the absence of language production requirements make the task child-friendly and suitable for a broad range of ages in childhood [47,49] as well as for children from different socioeconomic backgrounds. Moreover, this study provides evidence for the use of the task in at-risk populations, for example, children with lower general cognitive abilities, as has been found in children born preterm compared to term-born peers [93]. The task comprises a variety of different qualities with both positive and negative facets, reflected in concrete everyday activities and feelings (e.g., “never argue”, “argue a lot”), and considers the reciprocal nature of social relationships (i.e., items originated from the child and from the other person).

This study also has some limitations. First, studies that are more contemporary should replicate our findings, since data collection in this cohort was administered in the 1990s. However, other studies, which compared findings of the BLS with more recent cohorts, showed that outcomes did not differ according to time of measurement [54], and mothers’ and fathers’ roles in childrearing seem not to have significantly changed throughout the last decades [71]. Second, the prediction of children’s social relationship qualities accounted for a significant, however, modest amount of explained variance; thus, future studies should focus on other factors (e.g., characteristics of both persons in the dyadic relationship, environmental factors), which may contribute to children’s social relationship qualities across school transitions. Third, children might have associated several social relationships with a respective quality, but they were restricted to choosing only one social relationship in the used task [49]. However, the BLS tested different response formats, including rating scales, and found that the forced-choice format used in this study worked best, in particular considering that the BLS includes a sample of children born at neonatal risk with lower general cognitive ability [63] and children from different socioeconomic backgrounds. Moreover, this approach provides new insights into which social relationship is most important and first choice in satisfying certain social needs. Fourth, participants of this study came mostly from two-parent households and were German-speaking; however, there is evidence of cultural and family structure differences in children’s social relationship experiences [6,94,95]. Thus, the findings of this study exclusively apply to majority populations in Western industrialized countries, and future studies should investigate patterns of social relationship quality considering different ethnic groups and family structures [25].

## 5. Conclusions

This study provides unique insights on how children’s social relationships with parents, siblings, and friends develop from preschool to early school age. Overall, formal school entry significantly changes parents’, siblings’, and friends’ roles in fulfilling certain social needs in children’s daily lives. After school entry, children reported higher levels of parental comfort, perhaps due to the new challenges they have to cope with such as academic demands and growing peer groups. Moreover, although siblings spend a great deal of time together in their familial environment, friends seem to become increasingly important companions and preferred playmates. In contrast, conflict was found to represent a major facet in sibling relationships that increased from preschool to early school age. Given that sibling conflict and aggression is associated with maladjustment [29,30,85,96], early identification is crucial, and future studies should focus more on sibling relationships. Intra-individual stability in perceptions of social relationships over the two-year assessment period differed for social relationship qualities and types; average stability was low to modest. These results indicate that changes in social relationship qualities are a normative part of child development and formal school entry may represent an important junction.

## Figures and Tables

**Table 1:** Descriptive characteristics of the study sample (*N* = 1110).

Variables	*M* or *n*	*SD* or %
Child sex, female	554	(49.9%)
GA at birth, weeks	36.89	(3.75)
Born preterm (<37 weeks GA)	405	(36.5%)
Birth weight, grams	2727.35	(906.40)
Born with VLBW (<1500 g)	149	(13.4%)
Multiple births	88	(7.9%)
Neonatal risk ^a^		
None	294	(26.5%)
Low risk	211	(19.0%)
Moderate risk	224	(20.2%)
High risk	224	(20.2%)
Very high risk	155	(14.0%)
Family SES at birth		
High	374	(33.7%)
Middle	409	(36.8%)
Low	327	(29.5%)
Family status at birth, living together ^a^	1043	(94.7%)
Place of residence at birth ^a^		
Urban areas	359	(32.4%)
Rural areas	750	(67.6%)
Child age at 6-year assessment, years	6.21	(0.23)
Child age at 8-year assessment, years	8.34	(0.22)
School entry status at 6 years, in school	85	(7.7%)

*Notes*. Data are presented as mean (standard deviation) for continuous variables and numbers (percentage) for categorical variables. GA, gestational age; VLBW, very low birth weight; SES, socioeconomic status. ^a^ Descriptive statistics for Neonatal risk based on *n* = 1108 children, for Family status at birth on *n* = 1101 children, and for Place of residence at birth on *n* = 1109 children.

**Table 2:** Multivariate analyses of variance (MANOVAs) testing the effects of age on children’s social relationship quality with mothers, fathers, siblings, and friends (*N* = 1110).

Model	Pillai’s Trace	*F*	*df*	*p* ^a^	η*_p_*^2^
Age on social relationship quality with mothers	0.28	47.73	9, 1101	<0.001	0.28
Age on social relationship quality with fathers	0.14	19.47	9, 1101	<0.001	0.14
Age on social relationship quality with siblings	0.27	46.14	9, 1101	<0.001	0.27
Age on social relationship quality with friends	0.21	32.57	9, 1101	<0.001	0.21

*Notes*. ^a^ Two-tailed significance; models were adjusted for multiple testing using Bonferroni–Holm correction.

**Table 3:** Results of multiple linear regressions predicting valence scores at 8 years by valence scores at 6 years, separately for social relationship quality subscales and social relationship types (*N* = 1110).

Predictors at 6 Years	*R* ^2^	*F*	*B*	β	*t*	*BCa* 95% *CI* ^a^ for *B*
**Mother**						
Care	0.03	5.10 ***	0.12	0.14	4.70 **	[0.07, 0.18]
Comfort	0.06	11.17 ***	0.19	0.20	6.90 **	[0.14, 0.25]
Bad Conscience/Trust	0.05	9.03 ***	0.25	0.21	7.11 **	[0.18, 0.32]
Cuddling	0.07	14.05 ***	0.25	0.25	8.37 **	[0.19, 0.31]
Affection	0.04	6.92 ***	0.20	0.18	6.12 **	[0.14, 0.27]
Conflicts ^b^	0.01	2.21	0.08	0.07	2.33	[0.01, 0.14]
Fun/Fooling Around	0.02	3.11 *	0.08	0.09	2.87 *	[0.02, 0.14]
Playing	0.02	3.48 *	0.10	0.11	3.82 *	[0.04, 0.16]
Prosocial Behavior	0.01	2.08	0.06	0.09	2.84	[0.00, 0.12]
**Father**						
Care	0.03	6.40 ***	0.13	0.16	5.38 **	[0.08, 0.18]
Comfort	0.05	9.36 ***	0.17	0.19	6.34 **	[0.12, 0.23]
Bad Conscience/Trust	0.08	15.18 ***	0.30	0.26	8.87 **	[0.24, 0.37]
Cuddling	0.07	14.55 ***	0.22	0.21	7.04 **	[0.16, 0.28]
Affection	0.04	8.17 ***	0.19	0.17	5.71 **	[0.12, 0.26]
Conflicts ^b^	0.03	6.05 ***	0.16	0.15	5.15 **	[0.09, 0.23]
Fun/Fooling Around	0.02	3.25 *	0.11	0.11	3.60 **	[0.05, 0.17]
Playing	0.02	2.83	0.11	0.11	3.61	[0.05, 0.17]
Prosocial Behavior	0.01	2.60	0.09	0.11	3.51	[0.02, 0.16]
**Siblings**						
Care	0.05	9.91 ***	0.22	0.20	6.81 **	[0.15, 0.29]
Comfort	0.05	8.70 ***	0.21	0.18	6.15 **	[0.14, 0.28]
Bad Conscience/Trust	0.02	4.26 **	0.14	0.15	4.92 **	[0.07, 0.21]
Cuddling	0.03	5.10 ***	0.17	0.15	5.12 **	[0.10, 0.24]
Affection	0.08	16.89 ***	0.29	0.27	9.47 **	[0.22, 0.35]
Conflicts ^b^	0.08	16.78 ***	0.27	0.27	9.23 **	[0.21, 0.33]
Fun/Fooling Around	0.02	3.70 *	0.10	0.10	3.34 *	[0.04, 0.17]
Playing	0.06	12.53 ***	0.27	0.23	7.94 **	[0.20, 0.35]
Prosocial Behavior	0.06	10.79 ***	0.24	0.20	6.91 **	[0.16, 0.32]
**Friends**						
Care	0.03	5.27 ***	0.16	0.15	4.99 **	[0.09, 0.22]
Comfort	0.02	3.29 *	0.10	0.11	3.63 *	[0.04, 0.16]
Bad Conscience/Trust	0.01	2.10	0.05	0.07	2.22	[0.00, 0.10]
Cuddling	0.01	1.87	0.05	0.07	2.28	[0.00, 0.10]
Affection	0.03	4.75 **	0.12	0.13	4.27 **	[0.06, 0.17]
Conflicts ^b^	0.02	4.44 **	0.13	0.13	4.39 **	[0.07, 0.19]
Fun/Fooling Around	0.05	8.84 ***	0.14	0.14	4.85 **	[0.08, 0.20]
Playing	0.04	6.78 ***	0.16	0.15	5.01 **	[0.09, 0.22]
Prosocial Behavior	0.03	6.60 ***	0.15	0.15	5.03 **	[0.09, 0.20]

*Notes*. All models were adjusted for child sex, family socioeconomic status, gestational age, and school entry status at 6 years. *** *p* < 0.001, ** *p* < 0.01, * *p* < 0.05 (two-tailed), models were adjusted for multiple testing using Bonferroni–Holm correction. ^a^ 95% confidence intervals for unstandardized regression coefficients *B* are bias-corrected and accelerated, bootstrapping based on 5000 samples. ^b^ Higher values for the subscale Conflicts indicate lower frequency of conflicts; lower values indicate higher frequency of conflicts.

## Data Availability

De-identified summary data are available on request from the corresponding author due to local ethical and privacy restrictions.

## Supplementary Materials

The following are available online at https://www.mdpi.com/article/10.3390/children8100891/s1, Figure S1: Description of the Card-Sorting Task of the Friendship and Family Interview [65,66], Table S1: List of items of the Card-Sorting Task of the Friendship and Family Interview [65,66], Table S2: Correlations between valence scores used for companionship composite scores separately for social relationships with siblings and friends at 6 and 8 years (*N* = 1110), Table S3: Mean valence scores for social relationship quality subscales and social relationship types according to age at assessment (*N* = 1110).

## References

[1] Feiring C., Lewis M. (1991). The development of social networks from early to middle childhood: Gender differences and the relation to school competence. Sex Roles.

[2] Scott J.K., Nelson J.A., Dix T. (2018). Interdependence among mothers, fathers, and children from early to middle childhood: Parents’ sensitivity and children’s externalizing behavior. Dev. Psychol..

[3] Ladd G.W., Kochenderfer B.J., Coleman C.C. (1996). Friendship quality as a predictor of young children’s early school adjustment. Child Dev..

[4] Engle J.M., McElwain N.L., Lasky N. (2011). Presence and quality of kindergarten children’s friendships: Concurrent and longitudinal associations with child adjustment in the early school years. Infant. Child Dev..

[5] Yan J., Feng X., Schoppe-Sullivan S.J. (2018). Longitudinal associations between parent-child relationships in middle childhood and child-perceived loneliness. J. Fam. Psychol..

[6] McHale S.M., Updegraff K.A., Whiteman S.D. (2012). Sibling relationships and influences in childhood and adolescence. J. Marriage Fam..

[7] Furman W., Buhrmester D. (1985). Children’s perceptions of the personal relationships in their social networks. Dev. Psychol..

[8] Laursen B., Bukowski W.M. (1997). A developmental guide to the organisation of close relationships. Int. J. Behav. Dev..

[9] Rimm-Kaufman S.E., Pianta R.C. (2000). An ecological perspective on the transition to kindergarten: A theoretical framework to guide empirical research. J. Appl. Dev. Psychol..

[10] Lam C.B., McHale S.M., Crouter A.C. (2014). Time with peers from middle childhood to late adolescence: Developmental course and adjustment correlates. Child Dev..

[11] Rubin K.H., Coplan R.J., Chen X., Bowker J.C., MacDonald K.L., Heverly-Fitt S., Bornstein M.H., Lamb M.E. (2015). Peer relationships. Developmental Science: An Advanced Textbook.

[12] Perry K.E., Weinstein R.S. (1998). The social context of early schooling and children’s school adjustment. Educ. Psychol..

[13] Booth A., O’Farrelly C., Hennessy E., Doyle O. (2019). ‘Be good, know the rules’: Children’s perspectives on starting school and self-regulation. Childhood.

[14] Pike A., Oliver B.R. (2017). Child behavior and sibling relationship quality: A cross-lagged analysis. J. Fam. Psychol..

[15] Morrison E.F., Rimm-Kauffman S., Pianta R.C. (2003). A longitudinal study of mother-child interactions at school entry and social and academic outcomes in middle school. J. Sch. Psychol..

[16] Moss E., St-Laurent D. (2001). Attachment at school age and academic performance. Dev. Psychol..

[17] Shanahan L., McHale S.M., Osgood D.W., Crouter A.C. (2007). Conflict frequency with mothers and fathers from middle childhood to late adolescence: Within- and between-families comparisons. Dev. Psychol..

[18] Shanahan L., McHale S.M., Crouter A.C., Osgood D.W. (2007). Warmth with mothers and fathers from middle childhood to late adolescence: Within- and between-families comparisons. Dev. Psychol..

[19] De Goede I.H.A., Branje S.J.T., Meeus W.H.J. (2009). Developmental changes in adolescents’ perceptions of relationships with their parents. J. Youth Adolesc..

[20] Ainsworth M.S. (1989). Attachments beyond infancy. Am. Psychol..

[21] Bowlby J. (1982). Attachment and loss. Attachment.

[22] Kerns K.A., Tomich P.L., Kim P. (2006). Normative trends in children’s perceptions of availability and utilization of attachment figures in middle childhood. Soc. Dev..

[23] Driscoll K., Pianta R.C. (2011). Mothers’ and fathers’ perceptions of conflict and closeness in parent-child relationships during early childhood. J. Early Child. Infant Psychol..

[24] Dubois-Comtois K., Cyr C., Moss E. (2011). Attachment behavior and mother-child conversations as predictors of attachment representations in middle childhood: A longitudinal study. Attach. Hum. Dev..

[25] Seibert A.C., Kerns K.A. (2009). Attachment figures in middle childhood. Int. J. Behav. Dev..

[26] Furman W., Buhrmester D. (1992). Age and sex differences in perceptions of networks of personal relationships. Child Dev..

[27] Brody G.H. (2004). Siblings’ direct and indirect contributions to child development. Curr. Dir. Psychol. Sci..

[28] Hartup W.W., Stevens N. (1997). Friendships and adaptation in the life course. Psychol. Bull..

[29] Buist K.L., Vermande M. (2014). Sibling relationship patterns and their associations with child competence and problem behavior. J. Fam. Psychol..

[30] Wolke D., Tippett N., Dantchev S. (2015). Bullying in the family: Sibling bullying. Lancet Psychiatry.

[31] Gifford-Smith M.E., Brownell C.A. (2003). Childhood peer relationships: Social acceptance, friendships, and peer networks. J. Sch. Psychol..

[32] Tippett N., Wolke D. (2015). Aggression between siblings: Associations with the home environment and peer bullying. Aggress. Behav..

[33] Dunn J., Smith P.K., Hart C.H. (2002). Sibling relationships. Blackwell Handbook of Childhood Social Development.

[34] Howe N., Recchia H. (2005). Playmates and teachers: Reciprocal and complementary interactions between siblings. J. Fam. Psychol..

[35] Howe N., Aquan-Assee J., Bukowski W.M., Lehoux P.M., Rinaldi C.M. (2001). Siblings as confidants: Emotional understanding, relationship warmth, and sibling self-disclosure. Soc. Dev..

[36] Kosonen M. (1996). Siblings as providers of support and care during middle childhood: Children’s perceptions. Child Soc..

[37] Dantchev S., Wolke D. (2019). Trouble in the nest: Antecedents of sibling bullying victimization and perpetration. Dev. Psychol..

[38] Toseeb U., McChesney G., Dantchev S., Wolke D. (2020). Precursors of sibling bullying in middle childhood: Evidence from a UK-based longitudinal cohort study. Child Abuse Negl..

[39] Dunn J., Creps C., Brown J. (1996). Children’s family relationships between two and five: Developmental changes and individual differences. Soc. Dev..

[40] Nickerson A.B., Nagle R.J. (2005). Parent and peer attachment in late childhood and early adolescence. J. Early Adolesc..

[41] Kim J.-Y., McHale S.M., Wayne Osgood D., Crouter A.C. (2006). Longitudinal course and family correlates of sibling relationships from childhood through adolescence. Child Dev..

[42] Buhrmester D., Furman W. (1990). Perceptions of sibling relationships during middle childhood and adolescence. Child Dev..

[43] Cole A., Kerns K.A. (2001). Perceptions of sibling qualities and activities of early adolescents. J. Early Adolesc..

[44] De Goede I.H.A., Branje S.J.T., Meeus W.H.J. (2009). Developmental changes and gender differences in adolescents’ perceptions of friendships. J. Adolesc..

[45] Buhrmester D., Furman W. (1987). The development of companionship and intimacy. Child Dev..

[46] Updegraff K.A., McHale S.M., Crouter A.C. (2002). Adolescents’ sibling relationship and friendship experiences: Developmental patterns and relationship linkages. Soc. Dev..

[47] Boosman K., van der Meulen M., van Geert P., Jackson S. (2002). Measuring young children’s perceptions of support, control, and maintenance in their own social networks. Soc. Dev..

[48] Dunn J., Slomkowski C., Beardsall L. (1994). Sibling relationships from the preschool period through middle childhood and early adolescence. Dev. Psychol..

[49] Gleason T.R. (2002). Social provisions of real and imaginary relationships in early childhood. Dev. Psychol..

[50] Cutting A.L., Dunn J. (2006). Conversations with siblings and with friends: Links between relationship quality and social understanding. Br. J. Dev. Psychol..

[51] Jenkins J.M., Dunn J., O’Connor T.G., Rasbash J., Behnke P. (2005). Change in maternal perception of sibling negativity: Within- and between-family influences. J. Fam. Psychol..

[52] Raikes H.A., Virmani E.A., Thompson R.A., Hatton H. (2013). Declines in peer conflict from preschool through first grade: Influences from early attachment and social information processing. Attach. Hum. Dev..

[53] Tucker C.J., Finkelhor D., Shattuck A.M., Turner H. (2013). Prevalence and correlates of sibling victimization types. Child Abuse Negl..

[54] Wolke D., Baumann N., Strauss V., Johnson S., Marlow N. (2015). Bullying of preterm children and emotional problems at school age: Cross-culturally invariant effects. J. Pediatr..

[55] Ritchie K., Bora S., Woodward L.J. (2015). Social development of children born very preterm: A systematic review. Dev. Med. Child Neurol..

[56] Heuser K.M., Jaekel J., Wolke D. (2018). Origins and predictors of friendships in 6- to 8-year-old children born at neonatal risk. J. Pediatr..

[57] Potijk M.R., de Winter A.F., Bos A.F., Kerstjens J.M., Reijneveld S.A. (2015). Behavioural and emotional problems in moderately preterm children with low socioeconomic status: A population-based study. Eur. Child Adolesc. Psychiatry.

[58] Ritchie K., Bora S., Woodward L.J. (2018). Peer relationship outcomes of school-age children born very preterm. J.Pediatr..

[59] Korja R., Latva R., Lehtonen L. (2012). The effects of preterm birth on mother-infant interaction and attachment during the infant’s first two years. Acta Obstet. Gynecol. Scand..

[60] Mistry R.S., Vandewater E.A., Huston A.C., McLoyd V.C. (2002). Economic well-being and children’s social adjustment: The role of family process in an ethnically diverse low-income sample. Child Dev..

[61] Tippett N., Wolke D. (2014). Socioeconomic status and bullying: A meta-analysis. Am. J. Public Health.

[62] Riegel K., Ohrt B., Wolke D., Österlund K. (1995). Die Entwicklung gefaehrdet geborener Kinder bis zum fuenften Lebensjahr. Die Arvo Ylppoe Neugeborenen-Nachfolgestudie in Suedbayern und Suedfinnland. [The development of children born at risk until their fifth year of life. The Arvo Ylppoe Longitudinal Study in South Bavaria and South Finland.].

[63] Wolke D., Meyer R. (1999). Cognitive status, language attainment, and prereading skills of 6-year-old very preterm children and their peers: The Bavarian Longitudinal Study. Dev. Med. Child Neurol..

[64] Putnick D.L., Bornstein M.H., Eryigit-Madzwamuse S., Wolke D. (2017). Long-term stability of language performance in very preterm, moderate-late preterm, and term children. J. Pediatr..

[65] Wolke D. (1991). Manual zum Freundschafts- und Familieninterview. [Friendship and Family Interview.].

[66] Wolke D. (1993). Manual zum Freundschafts- und Familieninterview. 8-Jahres-Untersuchung. [Friendship and Family Interview. 8-Year Assessment.].

[67] Wolke D., Strauss V.Y.-C., Johnson S., Gilmore C., Marlow N., Jaekel J. (2015). Universal gestational age effects on cognitive and basic mathematic processing: 2 cohorts in 2 countries. J. Pediatr..

[68] Jaekel J., Pluess M., Belsky J., Wolke D. (2015). Effects of maternal sensitivity on low birth weight children’s academic achievement: A test of differential susceptibility versus diathesis stress. J. Child Psychol. Psychiatry.

[69] Wolke D., Jaekel J., Hall J., Baumann N. (2013). Effects of sensitive parenting on the academic resilience of very preterm and very low birth weight adolescents. J. Adolesc. Health.

[70] Bauer A. (1988). Ein Verfahren zur Messung des fuer das Bildungsverhalten relevanten Sozial Status (BRSS) - ueberarbeitete Fassung. [A measure assessing SES in Germany, revised version.].

[71] Craig L., Powell A., Smyth C. (2014). Towards intensive parenting? Changes in the composition and determinants of mothers’ and fathers’ time with children 1992-2006. Br. J. Sociol..

[72] Fox L., Han W.-J., Ruhm C., Waldfogel J. (2013). Time for children: Trends in the employment patterns of parents, 1967-2009. Demography.

[73] Statistisches Bundesamt Deutschland (Destatis) Abhängig Erwerbstätige: Deutschland, Jahre (bis 2019), Beschäftigungsumfang, Geschlecht. https://www-genesis.destatis.de/genesis/online?sequenz=tabelleErgebnis&selectionname=12211-9010#abreadcrumb.

[74] Cabrera N.J., Tamis-LeMonda C.S., Bradley R.H., Hofferth S., Lamb M.E. (2000). Fatherhood in the twenty-first century. Child Dev..

[75] Farmer T.W., McAuliffe Lines M., Hamm J.V. (2011). Revealing the invisible hand: The role of teachers in children’s peer experiences. J. Appl. Dev. Psychol..

[76] Elvert C., Johnson S., Jaekel J. (2021). Teachers’ knowledge and approaches to supporting preterm children in the classroom. Early Hum. Dev..

[77] Gest S.D., Rodkin P.C. (2011). Teaching practices and elementary classroom peer ecologies. J. Appl. Dev. Psychol..

[78] Conduct Problems Prevention Research Group (1999). Initial impact of the fast track prevention trial for conduct problems: II. classroom effects. J. Consult. Clin. Psychol..

[79] Webster-Stratton C., Reid M.J., Hammond M. (2004). Treating children with early-onset conduct problems: Intervention outcomes for parent, child, and teacher training. J. Clin. Child Adolesc. Psychol..

[80] Richardson H., Lisandrelli G., Riobueno-Naylor A., Saxe R. (2018). Development of the social brain from age three to twelve years. Nat. Commun..

[81] Quinn M., Hennessy E. (2010). Peer relationships across the preschool to school transition. Early Educ. Dev..

[82] Maccoby E.E. (1990). Gender and relationships: A developmental account. Am. Psychol..

[83] Campione-Barr N. (2017). The changing nature of power, control, and influence in sibling relationships. New Dir. Child Adolesc. Dev..

[84] Tucker C.J., Updegraff K.A., Baril M.E. (2010). Who’s the boss? Patterns of control in adolescents’ sibling relationships. Fam. Relat..

[85] Dantchev S., Hickman M., Heron J., Zammit S., Wolke D. (2019). The independent and cumulative effects of sibling and peer bullying in childhood on depression, anxiety, suicidal ideation, and self-harm in adulthood. Front. Psychiatry.

[86] Jaekel J., Wolke D., Chernova J. (2012). Mother and child behaviour in very preterm and term dyads at 6 and 8 years. Dev. Med. Child Neurol..

[87] Due P., Merlo J., Harel-Fisch Y., Damsgaard M.T., Holstein B.E., Hetland J., Currie C., Gabhainn S.N., de Matos M.G., Lynch J. (2009). Socioeconomic inequality in exposure to bullying during adolescence: A comparative, cross-sectional, multilevel study in 35 countries. Am. J. Public Health.

[88] Bilgin A., Wolke D. (2015). Maternal sensitivity in parenting preterm children: A meta-analysis. Pediatrics.

[89] Reyes L.M., Jaekel J., Kreppner J., Wolke D., Sonuga-Barke E. (2020). A comparison of the effects of preterm birth and institutional deprivation on child temperament. Dev. Psychopathol..

[90] Reyes L.M., Jaekel J., Bartmann P., Wolke D. (2021). Peer relationship trajectories in very preterm and term individuals from childhood to early adulthood. J. Dev. Behav. Pediatr..

[91] Bilgin A., Brylka A., Wolke D., Trower H., Baumann N., Lemola S. (2021). Subjective well-being and self-esteem in preterm born adolescents: An individual participant data meta-analysis. J. Dev. Behav. Pediatr..

[92] Mendonça M., Bilgin A., Wolke D. (2019). Association of preterm birth and low birth weight with romantic partnership, sexual intercourse, and parenthood in adulthood: A systematic review and meta-analysis. JAMA Netw. Open.

[93] Jaekel J., Scott M., Donders J., Hunter S.J. (2018). Preterm and low-birth-weight birth. Neuropsychological Conditions Across the Lifespan.

[94] O’Connor T.G., Dunn J., Jenkins J.M., Rasbash J. (2006). Predictors of between-family and within-family variation in parent-child relationships. J. Child Psychol. Psychiatry.

[95] French D.C., Rianasari M., Pidada S., Nelwan P., Buhrmester D. (2001). Social support of Indonesian and U.S. children and adolescents by family members and friends. Merrill-Palmer Q..

[96] Dantchev S., Zammit S., Wolke D. (2018). Sibling bullying in middle childhood and psychotic disorder at 18 years: A prospective cohort study. Psychol. Med..

