# Supplementary material for: The Impact of Formal School Entry on Children’s Social Relationships with Parents, Siblings, and Friends
Source: Children (Basel). 2021 Oct 6;8(10):891. doi: 10.3390/children8100891 (PMC8535132; doi:10.3390/children8100891)
Supplement: Supplementary file 1 [file children-08-00891-s001.zip › children-1347772-supplementary.pdf]

Supplementary Materials

Figure S1. Description of the Card-Sorting Task of the Friendship and Family Interview [65, 66].

Children's social relationships

Children were asked to list the people who belonged to their family or household and their playmates and friends:

- **Mother & Father:** Were included, even if children did not list them spontaneously (exception: child never knew parent or parent was dead), but were known to belong to the family. A new partner (stepparent or friend) was included, if parents were separated or divorced and the new partner slept over for more than two days a week, and was assigned to this category, if the biological parent was already dead and the new partner adopted the role of the mother or father.
- **Siblings:** All siblings (up to seven) living in the same household were included, even if children did not list them spontaneously, but were known to belong to the family.
- **Friends:** First five playmates or friends listed and up to five other best friends were included. Children who lived in the same household, but were not (step-) siblings (e.g., children in flat-sharing community, foster children) were considered as friends.

Children could also list grandparents and other related or non-related children and adults. However, these were not part of the analyses and are not reported here.

Setting

Children were instructed to choose Playmobil® figures that represented their mother, father, siblings, and friends. The Playmobil® figures were placed in groups on a board according to social relationship type that had a posting slit below each group.

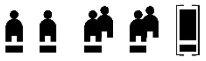

Quasi-randomization of item order

The Card-Sorting Task contained 36 cards each depicting a statement with a positive or negative feeling or action that had either originated from the child or from the other person (see Table S1).

- The task consists of **nine subscales**, each tapping into another social relationship quality or need with four items (i.e., two positive and two negative).

The order of items was quasi-randomized:

1. The relationship quality domains were mixed (i.e., *Parent-related*: Care, Comfort, Bad Conscience / Trust; *Peer-related*: Fun / Fooling Around, Playing, Prosocial Behavior; *Mixed*: Cuddling, Conflicts, Affection), but the task started with a positive quality (i.e., a) Cuddling, b) Care, c) Fun / Fooling Around, d) Conflicts, e) Comfort, f) Playing, g) Affection, h) Bad Conscience / Trust, and i) Prosocial Behavior).
2. The valence of the items (i.e., positive, negative) was mixed, but the task started with two positive cards, and then, was quasi-randomized (i.e., a) positive feeling / action originated from the child, b) negative feeling / action originated from the other person, c) positive feeling / action originated from the other person, and d) negative feeling / action originated from the child).

The described order was fixed for all participants (see Table S1 for card numbers).

Card sorting

The statements on the 36 cards were read out loud and reformulated as questions. Then, the children were asked to assign each respective card to one of the persons on the board using a forced-choice response format: (a) mother, (b) father, (c) siblings, or (d) friends. If they felt that a card did not apply to any of the available groups, it could be posted to a building block representing nobody that was also placed on the board.

- ***Interviewer:*** *"Look, this card states: 'With this person I always make excursions.' With whom of these persons do you always make excursions? To whom does the card fit best? … Then, you can post the card in the mailbox of …."*

Coding & Scoring

Responses for each card were coded numerically according to social relationship type:

- Mother = 1, father = 2, siblings = 3, friends = 4, nobody = 5, respectively.

Calculation of subscale valence scores:

- For each social relationship type, positive items were recoded into 1 (= positive card assigned to person) or 0 (= positive card assigned to other person), and negative items were recoded into −1 (= negative card assigned to person) or 0 (= negative card assigned to other person). For each social relationship type, responses were summed up across items separately for each of the nine subscales at 6 and 8 years of age.
- Valence scores ranged from −2 to +2, with higher values (i.e., positive values) indicating more positive than negative cards, lower values (i.e., negative values) indicating more negative than positive cards, and 0 indicating either no cards were assigned to this person, or the same number of positive and negative cards.

Table S1. List of items of the Card-Sorting Task of the Friendship and Family Interview [65, 66].

	Interview Questions / Items	Valence	Direction / Origin	Domain	Subscale
Card 2	"When you are ill, who looks after you?"	Positive	From the other person	Parent-related items	Care
Card 11	"When you are ill, who do you not want to see?"	Negative	From the child	Parent-related items	Care
Card 20	"When you are ill, who do you most like to be around?"	Positive	From the child	Parent-related items	Care
Card 29	"When you are ill, who does not care for you?"	Negative	From the other person	Parent-related items	Care
Card 5	"Who does not care when you are sad?"	Negative	From the other person	Parent-related items	Comfort
Card 14	"When you are sad, who comforts you?"	Positive	From the other person	Parent-related items	Comfort
Card 23	"When you are sad, who do you never go to?"	Negative	From the child	Parent-related items	Comfort
Card 32	"When you are sad, who do you always go to?"	Positive	From the child	Parent-related items	Comfort
Card 8	"When you did something wrong, who do you always go to?"	Positive	From the child	Parent-related items	Bad Conscience / Trust
Card 17	"When you did something wrong, who tells you off?"	Negative	From the other person	Parent-related items	Bad Conscience / Trust
Card 26	"Who is nice to you even when you did something wrong?"	Positive	From the other person	Parent-related items	Bad Conscience / Trust
Card 35	"When you did something wrong, who are you afraid to go to?"	Negative	From the child	Parent-related items	Bad Conscience / Trust
Card 3	"Who do you think is sometimes really boring?"	Negative	From the child	Peer-related items	Fun / Fooling Around
Card 12	"Who do you mess around with?"	Positive	From the child	Peer-related items	Fun / Fooling Around
Card 21	"Who does not mess around with you?"	Negative	From the other person	Peer-related items	Fun / Fooling Around
Card 30	"Who does always have fun ideas what to do?"	Positive	From the other person	Peer-related items	Fun / Fooling Around
Card 6	"Who likes to play with you?"	Positive	From the other person	Peer-related items	Playing
Card 15	"Who do you not at all like to play with?"	Negative	From the child	Peer-related items	Playing
Card 24	"Who can you play your favorite games with?"	Positive	From the child	Peer-related items	Playing
Card 33	"Who does not like to play with you?"	Negative	From the other person	Peer-related items	Playing
Card 9	"Who does not share with you?"	Negative	From the other person	Peer-related items	Prosocial Behavior
Card 18	"Who shares with you?"	Positive	From the other person	Peer-related items	Prosocial Behavior

Table S1. Continued.

	Interview Questions / Items	Valence	Direction / Origin	Domain	Subscale
Card 27	"Who do you not like to share your toys with?"	Negative	From the child	Peer-related items	Prosocial Behavior
Card 36	"Who do you like to share with, e.g., sweets?"	Positive	From the child	Peer-related items	Prosocial Behavior
Card 1	"Who do you most like to cuddle with?"	Positive	From the child	Mixed items	Cuddling
Card 10	"Who likes to cuddle with you?"	Positive	From the other person	Mixed items	Cuddling
Card 19	"Who do you not at all like to cuddle with?"	Negative	From the child	Mixed items	Cuddling
Card 28	"Who sends you away when you want to cuddle?"	Negative	From the other person	Mixed items	Cuddling
Card 4	"Who do you rarely argue with?"	Positive	From the child	Mixed items	Conflicts
Card 13	"Who does often start arguing with you?"	Negative	From the other person	Mixed items	Conflicts
Card 22	"Who does not start arguing with you?"	Positive	From the other person	Mixed items	Conflicts
Card 31	"Who do you argue a lot with?"	Negative	From the child	Mixed items	Conflicts
Card 7	"Who do you not like very much?"	Negative	From the child	Mixed items	Affection
Card 16	"Who do you like a lot?"	Positive	From the child	Mixed items	Affection
Card 25	"Who do you think does not like you?"	Negative	From the other person	Mixed items	Affection
Card 34	"Who likes you a lot?"	Positive	From the other person	Mixed items	Affection

Table S2. Correlations between valence scores used for companionship composite scores separately for social relationships with siblings and friends at 6 and 8 years (N = 1110).

		Social Relationship Type					
		**Siblings**			**Friends**		
	**Subscale**	**1**	**2**	**3**	**1**	**2**	**3**
	**6-Year Assessment**						
1	Fun / Fooling Around	-			-		
2	Playing	0.389	-		0.395	-	
3	Prosocial Behavior	0.353	0.500	-	0.369	0.512	-
	**8-Year Assessment**						
1	Fun / Fooling Around	-			-		
2	Playing	0.461	-		0.449	-	
3	Prosocial Behavior	0.379	0.590	-	0.432	0.521	-

Notes. Data is presented as Pearson correlation coefficients r. All correlations were significant at $p < 0.001$ (two-tailed); models were adjusted for multiple testing using Bonferroni–Holm correction.

Table S3. Mean valence scores for social relationship quality subscales and social relationship types according to age at assessment (N = 1110).

	Age at Assessment					
	6 Years		8 Years			
**Subscales**	***M***	***SD***	***M***	***SD***	F [a]	η_p^2
**Mother**						
Care	0.96	(0.74)	1.34	(0.65)	184.93***	0.14
Comfort	0.80	(0.86)	1.19	(0.82)	145.69***	0.12
Bad Conscience / Trust	−0.08	(0.98)	0.30	(1.17)	86.49***	0.07
Cuddling	0.64	(0.88)	0.94	(0.88)	88.02***	0.07
Affection	0.40	(0.72)	0.66	(0.81)	75.93***	0.06
Conflicts [b]	0.16	(0.51)	0.22	(0.55)	9.56*	0.01
Fun / Fooling Around	0.00	(0.53)	−0.10	(0.50)	19.44***	0.02
Playing	0.11	(0.51)	0.11	(0.44)	0.01	0.00
Prosocial Behavior	0.15	(0.52)	0.09	(0.36)	11.89**	0.01
**Father**						
Care	0.03	(0.69)	0.10	(0.57)	8.32*	0.01
Comfort	0.06	(0.73)	0.27	(0.67)	59.42***	0.05
Bad Conscience / Trust	−0.39	(0.91)	−0.54	(1.08)	14.90**	0.01
Cuddling	0.18	(0.79)	0.32	(0.84)	21.93***	0.02
Affection	0.17	(0.69)	0.41	(0.77)	67.65***	0.06
Conflicts [b]	0.07	(0.55)	0.19	(0.58)	29.11***	0.03
Fun / Fooling Around	−0.05	(0.64)	−0.05	(0.66)	0.00	0.00
Playing	0.05	(0.56)	0.05	(0.57)	0.06	0.00
Prosocial Behavior	0.03	(0.49)	0.06	(0.41)	2.39	0.00
**Siblings**						
Care	−0.23	(0.82)	−0.40	(0.88)	29.57***	0.03
Comfort	−0.19	(0.82)	−0.80	(0.94)	326.22***	0.23
Bad Conscience / Trust	0.12	(0.60)	0.10	(0.56)	0.31	0.00
Cuddling	−0.09	(0.86)	−0.24	(0.96)	16.56***	0.01
Affection	−0.17	(0.87)	−0.36	(0.92)	34.25***	0.03
Conflicts [b]	−0.41	(1.01)	−0.88	(1.03)	162.28***	0.13
Fun / Fooling Around	−0.05	(0.85)	−0.24	(0.88)	30.23***	0.03
Playing	−0.08	(0.89)	−0.20	(1.03)	11.38**	0.01
Prosocial Behavior	−0.05	(0.99)	−0.13	(1.17)	4.05	0.00
**Friends**						
Care	−0.19	(0.84)	−0.29	(0.87)	8.69*	0.01
Comfort	−0.32	(0.86)	−0.33	(0.79)	0.01	0.00
Bad Conscience / Trust	0.39	(0.73)	0.27	(0.58)	22.64***	0.02
Cuddling	−0.13	(0.86)	−0.31	(0.66)	33.55***	0.03
Affection	0.18	(1.11)	0.06	(1.03)	8.16*	0.01
Conflicts [b]	0.51	(1.06)	0.59	(1.05)	4.16	0.00
Fun / Fooling Around	0.42	(1.01)	0.82	(1.01)	100.66***	0.08
Playing	0.63	(1.04)	0.81	(1.08)	19.55***	0.02
Prosocial Behavior	0.42	(1.11)	0.76	(1.08)	63.30***	0.05

Notes. Data is presented as mean (*SD*). All scores ranged from −2 to +2. *** $p < 0.001$, ** $p < 0.01$, * $p < 0.05$, models were adjusted for multiple testing using Bonferroni−Holm correction. [a] Results of repeated-measures one-way ANOVAs with age (6 years vs. 8 years) as within-subjects factor. [b] Higher values for the subscale Conflicts indicate lower frequency of conflicts; lower values indicate higher frequency of conflicts.
